# Circulating MicroRNAs and Novel Proteins as Potential Biomarkers of Neurological Complications after Heart Bypass Surgery

**DOI:** 10.3390/jcm10143091

**Published:** 2021-07-13

**Authors:** Krzysztof Szwed, Magdalena Szwed, Mariusz Kozakiewicz, Joanna Karłowska-Pik, Natalia Soja-Kukieła, Adrianna Bartoszewska, Alina Borkowska

**Affiliations:** 1 Department of Clinical Neuropsychology, Collegium Medicum, Nicolaus Copernicus University, 87-100 Toruń, Poland; szwed.fm@gmail.com (M.S.); alab@cm.umk.pl (A.B.); 2 Department of Geriatrics, Divison of Biochemistry and Biogerontology, Collegium Medicum, Nicolaus Copernicus University, 87-100 Toruń, Poland; markoz@cm.umk.pl (M.K.); a.baroszewska@cm.umk.pl (A.B.); 3 Department of Mathematical Statistics and Data Mining, Nicolaus Copernicus University, 87-100 Toruń, Poland; joanka@mat.umk.pl; 4 Centre for Statistical Analysis, Nicolaus Copernicus University, 87-100 Toruń, Poland; natalia.sk@umk.pl

**Keywords:** microRNA, biomarker, neurological complications, delirium, postoperative cognitive dysfunction, POCD, cardiac surgery, coronary artery bypass grafting, CABG, clinical study

## Abstract

Postoperative recovery can be impaired by many conditions, some of which are difficult to diagnose clinically. These include type 2 neurological complications such as hypoactive subtype of postoperative delirium (PD) and early postoperative cognitive dysfunction (ePOCD). Hope for their timely detection may lie with novel biomarkers. Plasma concentrations of microRNA-1-3p, microRNA-21-5p, glial fibrillary acidic protein (GFAP), neuroserpin (NSP), phosphorylated axonal neurofilament subunit H (pNfH) and visinin-like protein 1 (VILIP-1) were investigated in 30 patients undergoing elective off-pump coronary artery bypass grafting. Blood samples were collected at the start and end of a surgery as well as 24 h postoperatively. Associations between the studied biomarkers’ perioperative expression and type 2 neurological complications were analyzed. PD was associated with postoperative expression of GFAP; ePOCD was associated with postoperative expression of microRNA-21-5p and GFAP as well as intraoperative expression of NSP. The predictive accuracy of these molecules was found acceptable, with all their areas under the curve (AUC) values above 0.7. Multivariable regression indicated that microRNA-21-5p, GFAP and NSP were the only significant predictors of ePOCD. Evaluation of a multi-marker model including these three molecules revealed its outstanding predictive accuracy for ePOCD (AUC = 0.95). The use of microRNA-21-5p, GFAP and NSP for monitoring postoperative recovery warrants further research considering their potential to predict PD and ePOCD.

## 1. Introduction

Heart bypass surgery is one of the most widely performed major surgeries in the world. Despite the introduction of neuroprotective operating techniques such as the off-pump coronary artery bypass (OPCAB) method and its state-of-the-art modifications, recovery from this procedure is still frequently impaired by neurological complications [1]. These have a wide range of manifestations and can be categorized into either type 1, which is associated with major focal deficits, or type 2, which includes postoperative delirium (PD) and early postoperative cognitive dysfunction (ePOCD) [2]. It should be noted that the significant negative impact of PD and ePOCD is further magnified by their underdiagnosis [3]. Therefore, identifying a biochemical method to reliably predict these problems would be of remarkable clinical value.

Promising candidate biomarkers of PD and ePOCD can be found among proteins such as glial fibrillary acidic protein (GFAP), neuroserpin (NSP), phosphorylated axonal neurofilament subunit H (pNfH) and visitin-like protein 1 (VILIP-1). They are very specific to brain injury, highly sensitive and easily detectable in venous blood [4,5,6,7]. Nevertheless, proteins are not the only molecules with the potential to detect type 2 neurological complications. MicroRNAs are small non-coding RNA molecules that regulate the gene expression. They have been proposed as clinical biomarkers for many pathological processes including heart injury, which is associated with microRNA-1 and microRNA-21 [8]. In addition, increased plasma concentrations of microRNA-1 showed diagnostic potential for myocardial injury in adult cardiac surgery [9] and the ability to predict cardiopulmonary bypass-related ischemic complications in pediatric heart surgery [10]. Meantime, low preoperative serum concentrations of microRNA-21 were linked to acute kidney injury after adult cardiac surgery [11], while its increased plasma concentrations showed promise in diagnosing acute stroke as well as distinguishing its phases [12]. Nevertheless, none of these microRNAs have yet been studied in the context of PD and ePOCD prediction.

The main aim of this research was to investigate microRNA-1-3p, microRNA-21-5p, GFAP, NSP, pNfH and VILIP-1 as candidate biomarkers of PD and ePOCD.

## 2. Materials and Methods

### 2.1. Study Design

This study was a single-center assessor- and patient-blinded case–control study.

### 2.2. Setting

This study was conducted at the Department of Cardiac Surgery, Dr Antoni Jurasz Memorial University Hospital, Bydgoszcz, Poland. This tertiary care center performs over 500 OPCAB annually.

### 2.3. Participants

This study’s participants were recruited from patients scheduled for elective isolated first-time OPCAB. They were not eligible if they presented any of the following conditions: neurological or psychiatric disorders, alcohol or drug abuse, preoperative left ventricular ejection fraction of less than 30% or extracranial carotid artery stenosis of more than 70%. Additionally, each candidate was screened for dementia and mood disorders with Mini-Mental State Examination (MMSE) and the Hospital Anxiety and Depression Scale (HADS). Scoring below age- and education-adjusted cut-off values in MMSE resulted in exclusion from this research, while scoring over seven points on the sub-scales of HADS entailed a psychiatric consultation to verify patient’s eligibility.

All patients underwent OPCAB through a median sternotomy using the Octopus Medtronic coronary stabilizer for distal anastomosis and a side-biting clamp on the aorta for proximal anastomosis. The skeletonized left internal mammary artery was used for grafting all left anterior descending coronary artery lesions and either the skeletonized mammary artery or saphenous vein grafts were used for all others. The operators were qualified specialists who performed at least 500 OPCAB procedures before joining this research. All interventions in this study were completed under the same anaesthetic protocol (induction—fentanyl, etomidate; maintenance—propofol, sevoflurane, fentanyl). All patients were treated before and after surgery according to the current European Society of Cardiology Guidelines.

### 2.4. Variables

This study’s cases were selected based on the diagnosis of either PD or ePOCD and compared with controls selected based on the absence of the complication in question. This study’s exposures were the intra- and postoperative expressions of microRNA-1-3p, microRNA-21-5p, NSP, GFAP, pNfH and VILIP-1. Intraoperative expression was defined as the end to start of surgery ratio of a candidate biomarker’s plasma levels. Postoperative expression was defined as 24 h postoperative to the end of surgery ratio of a candidate biomarker’s plasma levels.

### 2.5. Data Sources/Measurement

Postoperative delirium was diagnosed within 7 days after surgery using Confusion Assessment Method for the Intensive Care Unit [13]. Screening commenced 24 h after OPCAB and was performed twice daily at 08:00 and 20:00 h for 6 days.

Early postoperative cognitive dysfunction was diagnosed on day 7 after surgery if a decline from preoperative performance (measured 2 days before the operation) was more than 20% in 2 or more postoperative tests. The cognitive test battery included 4 well-established instruments: Stroop Test, Trail Making Test, Digit Span Test and Rey Auditory Verbal Learning Test.

MicroRNAs and proteins were measured in samples acquired at the start and end of surgery as well as 24 h postoperatively. At these time points, whole blood was drawn from the ulnar vein into 10 mL anticoagulation tubes containing ethylene diamine tetraacetic acid. After 1 h it was centrifuged for 20 min at 2000 *g* in 4 °C. Subsequently plasma was divided into 200 µL aliquots and stored at −80 °C until analysis. The BioVendor kits (Laboratorni medicina a.s. Brno, Czech Republic) were used to estimate the concentration of GAFP, NSP, pNfH, VILIP-1, hsa-miR-1-3p (RDM0019H) and hsa-miR-21-5p (RDM0001H). All methods are enzyme-calibrated immunoassays with inner control. The methods for microRNA quantification involve hybridization of selected microRNA isolated from a patient’s sample, to a complementary biotinylated DNA probe for selected microRNA. The concentration of selected microRNA was determined by measuring the absorbance. A standard curve was constructed for each measured parameter by plotting the absorbance values against the standard concentrations for the selected parameters. Concentrations of unknown samples and Quality Control were determined using this standard curve. Synthetic nonhuman RNA Spike-In Control cel-miR-39-3p miREIA was added to the samples after the addition of lysis buffer for monitoring the efficiency of both microRNA molecules’ isolation. The concentration of Spike-In Control in samples were measured by using a DNA probe for Spike-In Control in parallel with the concentration of selected microRNA. To calculate the coefficient of isolation efficiency, the defined amount added to the samples before isolation is divided by the concentration of Spike-In Control measured by miREIA. Finally, the concentration of selected microRNA measured by miREIA was multiplied by the coefficient of isolation efficiency for every sample. Since miREIA is a relatively new method of microRNA detection, 30% of samples were also tested by a quantitative reverse transcription polymerase chain reaction (RT-qPCR). This showed a 99.7% correlation between the results of these methods.

### 2.6. Statistical Analysis

Most of the analyses were performed with PS IMAGO PRO (version 7) statistical software based on the IBM SPSS Statistics (version 27) analytical engine. Univariable logistic regression was performed with the R statistical software (version 4.0.2). Categorical variables were reported as numbers and percentages. Continuous variables were reported as either mean ± standard deviation or median and interquartile ranges according to their distribution, as assessed by the Shapiro–Wilk test. Results of all repeated measures were analyzed with Friedman’s exact test due to non-normal distribution. The strength of linear and monotonic correlations between biomarkers and other continuous variables was examined using Pearson and Spearman correlation coefficients, respectively. Pairwise between-group comparisons were analyzed using Mann–Whitney U test due to non-normal distributions of most variables. If the distributions were normal, a Student’s *t*-test was additionally performed. If between-group differences within the same measure were statistically significant, ROC curve analysis was performed. For PD and ePOCD prediction, univariable and multivariable logistic regression (with forward variable selection) models were built. Statistical significance was established at a *p*-value of <0.05 and Bonferroni correction was applied for multiple comparisons. All calculations were made using the database available as Supplementary data.

## 3. Results

### 3.1. Participants

Thirty-five patients were examined for eligibility. Of these, four did not meet the inclusion criteria and one refused to participate; the remaining 30 were enrolled and analyzed without any missing data.

### 3.2. Descriptive Data

Demographic and clinical data of study participants are reported in Table 1.

### 3.3. Main Results

**Figure 1:**
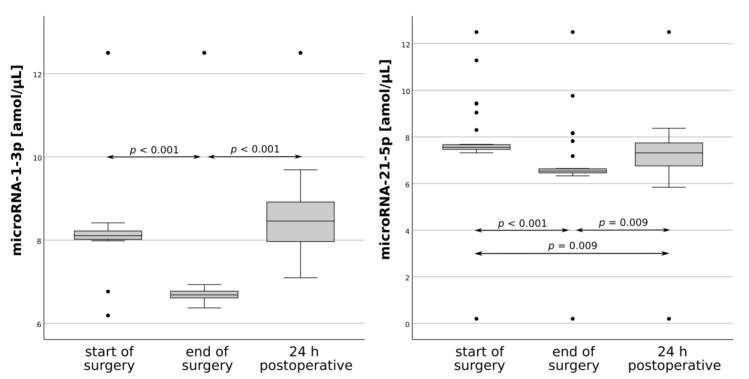
Time course of microRNA-1-3p and microRNA-21-5p plasma levels. The lower boundary of the box indicates the 25th percentile; the upper boundary of the box indicates the 75th percentile; the line within the box marks the median. Whiskers indicate 10th and 90th percentiles, and dots represent outliers.

Firstly, the effect of surgery on candidate biomarkers levels was examined. MicroRNA-1-3p and microRNA-21-5p had significantly different expression levels after OPCAB (Figure 1). Conversely, neither protein showed clear changes in perioperative levels, although differences in NSP concentrations were significant in the omnibus test but not in the post hoc tests. Next, the prognostic value of candidate biomarkers was explored by associating changes in their intra- and postoperative expression with various postoperative complications. This revealed that PD was associated with postoperative GFAP, while ePOCD was associated with postoperative microRNA-21-5p and GFAP as well as intraoperative NSP (Table 2 and Supplementary Figures S1–S3).

### 3.4. Other Analyses

**Figure 2:**
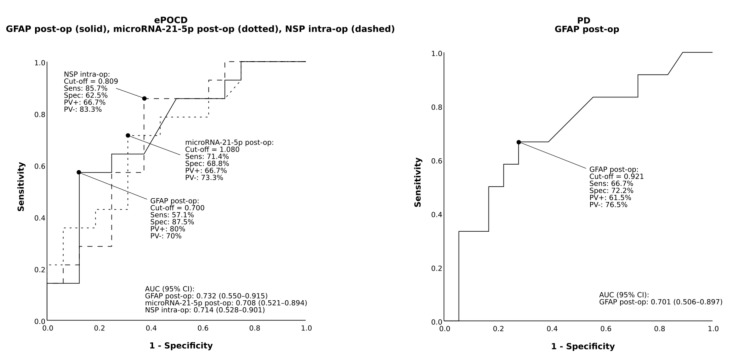
Receiver operating characteristic of postoperative expression (i.e., 24 h postoperative to end of surgery plasma level ratio [post-op]) of microRNA-21-5p and glial fibrillary acidic protein (GFAP), as well as intraoperative expression (i.e., end to start of surgery plasma level ratio [intra-op]) of neuroserpin (NSP) to predict postoperative delirium (PD) and early postoperative cognitive dysfunction (ePOCD). AUC indicates area under the curve; CI, confidence interval; PV+, positive predictive value; PV-, negative predictive value; Sens, sensitivity; Spec, specificity.

Candidate biomarkers’ predictive performances were also assessed through receiver operating characteristic (ROC) and the area under the curve (AUC) analysis. For predicting PD the AUC value of postoperative GFAP was found to be acceptable at 0.701. Similarly, for predicting ePOCD, the AUC values of postoperative microRNA-21-5p and GFAP, as well as intra-operative NSP, were also found to be acceptable at 0.708, 0.732 and 0.714, respectively (Figure 2).

**Figure 3:**
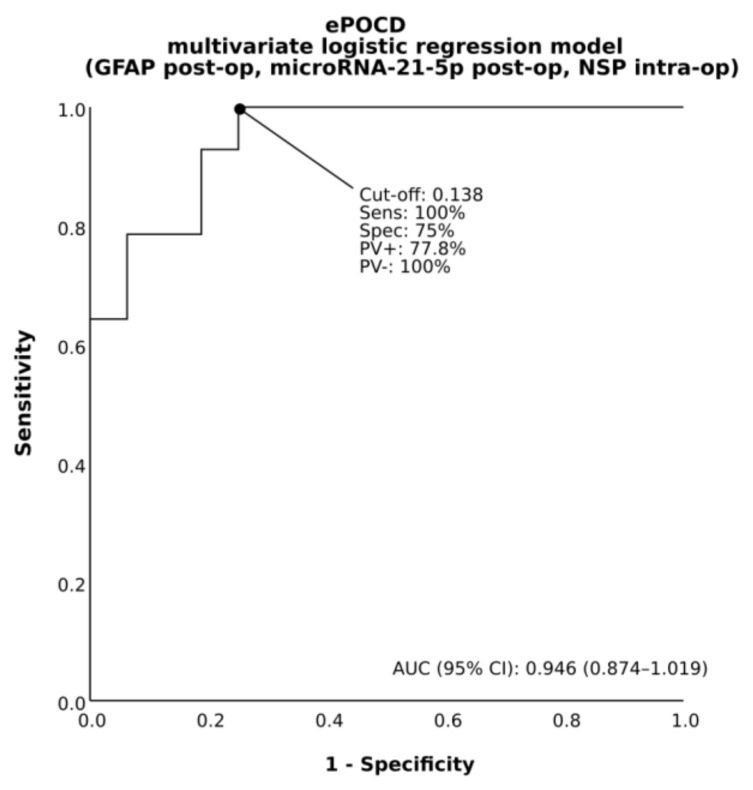
Receiver operating characteristic of a multivariable logistic regression model (incorporating the value of postoperative expression (i.e., 24 h postoperative to end of surgery plasma level ratio) of microRNA-21-5p and glial fibrillary acidic protein [GFAP] as well as an intraoperative expression (i.e., end to start of surgery plasma level ratio) of neuroserpin [NSP]) to predict early postoperative cognitive dysfunction (ePOCD). AUC indicates area under the curve; CI, confidence interval; PV+, positive predictive value; PV-, negative predictive value; Sens, sensitivity; Spec, specificity.

In addition, univariable and multivariable logistic regression was used to identify predictors of type 2 neurological complications. There were no characteristics significantly associated with PD in univariable analyses. Since no variables met the criterion, a multivariable model was not completed for this outcome. In univariable analysis, only microRNA-21-5p was significantly associated with ePOCD. In multivariable analysis, microRNA-21-5p, GFAP and NSP were significantly associated with this complication (Table 3). In the final analysis for predicting ePOCD, the AUC value of a multivariable logistic regression model was found to be outstanding at 0.946 (Figure 3).

## 4. Discussion

Type 2 neurological complications are most likely caused by cerebral ischemia and neuroinflammation [14]. Taking into account the role of the study’s biomarkers in these pathological pathways may elucidate their association with the development of PD and ePOCD. Notably, microRNA-21-5p was previously related to both ischemic stroke [12] and the inflammatory response to spinal cord injury [15]. In addition, NSP was also shown to play an important role in cerebral ischemia [16], while GFAP was proposed as a sensitive biomarker for the differentiation of ischemic and haemorrhagic stroke [17]. Therefore, it is probable that microRNA-21-5p, GFAP and NSP may predict type 2 neurological complications by early and accurate diagnosis of their low-key triggering processes.

This is the first report on the potential usefulness of microRNA-21-5p in predicting ePOCD. Importantly, it was recognized in all stages of statistical analysis, which is a promising result since microRNA molecules are inherently good biomarker candidates due to their small size and stability. Meanwhile, GFAP is used as a candidate biomarker in several ongoing clinical trials. Although it was shown to predict PD and ePOCD in this study, as well as neurodevelopmental outcomes in neonates who have undergone cardiac surgery [18], there are publications that challenge its usefulness [19,20]. In contrast, NSP is a completely new candidate biomarker for cardiac surgery and was only assessed in this clinical setting once—in our own preliminary research [19]. There it demonstrated an ability to predict type 2 neurological complications, which was also observed in the present study. This consistency of outcomes encourages further investigation into NSP’s utility. It should also be emphasized that using a multi-marker approach is essential when examining disorders of such complex pathophysiology as PD and ePOCD. This is illustrated by the current study’s three-molecule ePOCD predictive model being far more accurate than either of the molecules alone. Lastly, although the results of this report suggest that microRNA-1-3p, pNfH and VILIP-1 may not be suitable for predicting PD and ePOCD, they could still be appropriate for monitoring other aspects of postoperative recovery. In particular, microRNA-1-3p’s role is worth exploring due its association with this study’s data reflecting the extent of surgery (number of grafts) and its burden (time of intubation and hospitalization).

In related investigations increased plasma concentrations of microRNA-1-3p and microRNA-21-5p were associated with adverse consequences of heart surgery and stroke [9,10,12] which is in line with this study’s results. Previous data was obtained with RT-qPCR while ours was obtained with miREIA. The miREIA technique is fast, specific and has great correlation with RT-qPCR [21]. Importantly, it enables easy application of microRNA biomarkers in clinical laboratories that do not have a PCR machine.

Subsequent studies can use these exploratory findings as a benchmark to formulate specific hypotheses and calculate appropriate sample sizes for their testing. In order to improve generalizability, future research may consider examining patients with baseline neuropsychiatric disorders, who were excluded from this study. Moreover, forthcoming experiments could benefit from focusing on the neuroinflammatory pathophysiological pathways of type 2 neurological complications. Finally, this type of investigation could be extended to on-pump heart bypass grafting and other invasive medical procedures, since PD and ePOCD are also common outside of cardiac surgery.

## Figures and Tables

**Table 1:** Demographic and clinical characteristics of study participants.

Characteristic		Total *n* = 30
Age, y		66 (63–71)
Female sex		8 (26.7%)
Recent myocardial infarction (≤90 d)		10 (33.3%)
Hypertension		25 (83.3%)
Diabetes		10 (33.3%)
Creatinine clearance, mL/min		92.0 ± 21.5
LVEF, %		66.2 ± 9.6
BMI, kg/m^2^		28.1 ± 4.3
CCS class 4 angina		5 (16.7%)
NYHA class	I	1 (3.3%)
	II	1 (3.3%)
	III	2 (6.7%)
	IV	0
Logistic Euroscore, %		0.84 (0.67–1.01)

Values are presented as mean ± standard deviation, median (interquartile range) or *n* (%). BMI indicates body mass index; CCS, Canadian Cardiovascular Society; LVEF, left ventricle ejection fraction; NYHA, New York Heart Association.

**Table 2:** Associations of candidate biomarkers’ intra- and postoperative expression changes with perioperative characteristics.

Characteristic		Total *n* = 30	microRNA-1-3p		microRNA-21-5p		GFAP		pNfH		NSP		VILIP-1	
			Intra-Op	Post-Op	Intra-Op	Post-Op	Intra-Op	Post-Op	Intra-op	Post-op	Intra-op	Post-op	Intra-Op	Post-Op
Time of intubation, min		609.5 ± 146.6	r = −0.295 (*p* = 0.114) rho = −0.409 (*p* = 0.025)					r = −0.266 (*p* = 0.155) rho = −0.399 (*p* = 0.029)					r = 0.114 (*p* = 0.550) rho = 0.396 (*p* = 0.030)	
Surgery time		213.8 ± 49.0												
Grafts	1	1 (3.3%)		□ 1.194 ± 0.125 ● 1.271 ± 0.093 *p* = 0.060 *^,^^†^										
	2	12 (40%)												
	3	15 (50%)												
	4	2 (6.7%)												
Prolonged ICU stay (>48 h)		7 (23.3%)						□ 1.000 (0.852–1.342) ● 0.689 (0.511–0.691) *p* = 0.005					□ 0.918 (0.750–1.065) ● 1.200 (1.000–5.889) *p* = 0.021	
Prolonged hospitalization (>7 d)		12 (40%)		□ 1.211 ± 0.100 ● 1.277 ± 0.124 *p* = 0.117 *							□ 0.816 (0.575–0.848) ● 1.051 (0.802–1.520) *p* = 0.031			
PD		12 (40%)						□ 1.000 (0.852–1.342) ● 0.699 (0.617–1.000) *p* = 0.066 **						
Stroke		1 (3.3%)												
Transient ischemic attack		0												
ePOCD		14 (46.7%)				□ 1.027 ± 0.112 ● 1.113 ± 0.082 *p* = 0.025		□ 1.024 (0.905–1.571) ● 0.691 (0.665–1.000) *p* = 0.029			□ 0.800 (0.544–1.032) ● 0.874 (0.823–1.284) *p* = 0.047			
All-cause mortality		0												
Major bleeding		0												
Myocardial infarction		1 (3.3%)												
Deep sternal wound infection		3 (10%)		□ 1.223 ± 0.107 ● 1.367 ± 0.090 *p* = 0.035										
Atrial fibrillation		6 (20%)						□ 1.000 (0.700–1.145) ● 0.649 (0.511–0.884) *p* = 0.042						
Acute kidney injury		4 (13.3%)												

Values are presented as mean ± standard deviation, median (interquartile range) or *n* (%). Controls and cases are denoted by □ and ●, respectively. ePOCD indicates early postoperative cognitive dysfunction; GFAP, glial fibrillary acidic protein; ICU, intensive care unit; intra-op, intraoperative expression (end to start of surgery plasma level ratio); IQR, interquartile range; MAP, mean arterial pressure; NSP, neuroserpin; PD, postoperative delirium; pNfH, phosphorylated axonal neurofilament subunit H (pNfH); post-op, postoperative expression (24 h postoperative to end of surgery plasma level ratio); RCC, red cell concentrate; VILIP-1, visitin-like protein 1. * These differences were statistically significant during the previous step of the analysis (comparing medians) with *p* = 0.047 for “Grafts” and *p* = 0.049 for “Prolonged hospitalization”. ** Due to the borderline statistical significance of this variable, we performed an additional analysis comparing patients with PD to patients without any neurological complications (i.e., ePOCD cases, that could have influenced the outcome, were excluded from the control group) which resulted in *p* = 0.007. ^†^ For this, variable cases were defined as having either 3 or 4 grafts, while controls were defined as having either 1 or 2 grafts.

**Table 3:** Multivariable logistic regression of predictors for ePOCD.

Variable in Equation	B	*p*-Value	OR	95% CI for OR	
				Upper	Lower
GFAP post-op	−4.8	0.045	8.1 × 10^−3^	7.4 × 10^−5^	0.9
microRNA-21-5p post-op	20.1	0.027	5.5 × 10^−8^	10.3	2.9 × 10^16^
NSP intra-op	2.8	0.031	16.4	1.3	209.1
intercept	−19.9	0.051			

CI indicates confidence interval; GFAP, glial fibrillary acidic protein; intra-op, intraoperative expression (i.e., end to start of surgery plasma level ratio); NSP, neuroserpin; OR, odds ratio; post-op, postoperative expression (i.e., 24 h postoperative to end of surgery plasma level ratio).

## Data Availability

The data presented in this study are available in the supplementary material.

## Supplementary Materials

Graphical presentation of the study’s results as well as its database are available online at https://www.mdpi.com/article/10.3390/jcm10143091/s1. Supplementary Material 1: Study’s database; Figure S1: Scatter plots with fit lines, Figure S2: Bar charts comparing means in groups, with bar errors representing the variable’s standard deviation in groups, Figure S3: Box plots comparing variable’s distribution in groups.
