# Supplementary material for: Circulating MicroRNAs and Novel Proteins as Potential Biomarkers of Neurological Complications after Heart Bypass Surgery
Source: J Clin Med. 2021 Jul 13;10(14):3091. doi: 10.3390/jcm10143091 (PMC8303535; doi:10.3390/jcm10143091)
Supplement: Supplementary file 1 [file jcm-10-03091-s001.zip › jcm-1252946-supplementary 2-final.pdf]

Supplementary Material 2

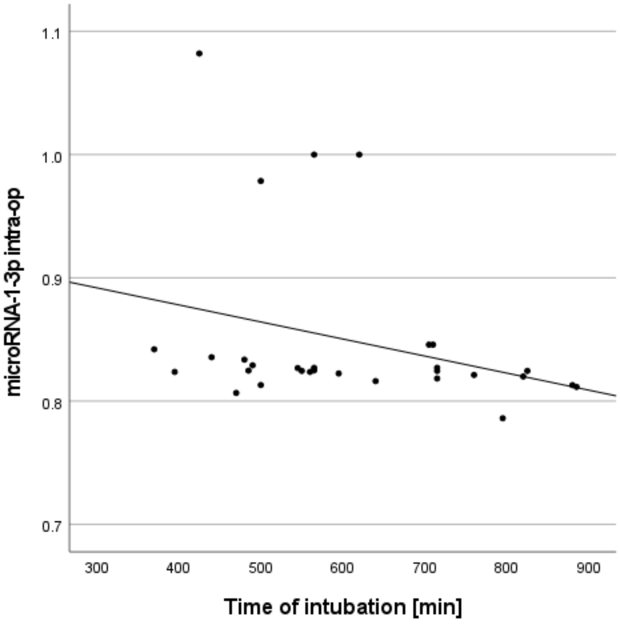

(**a**)

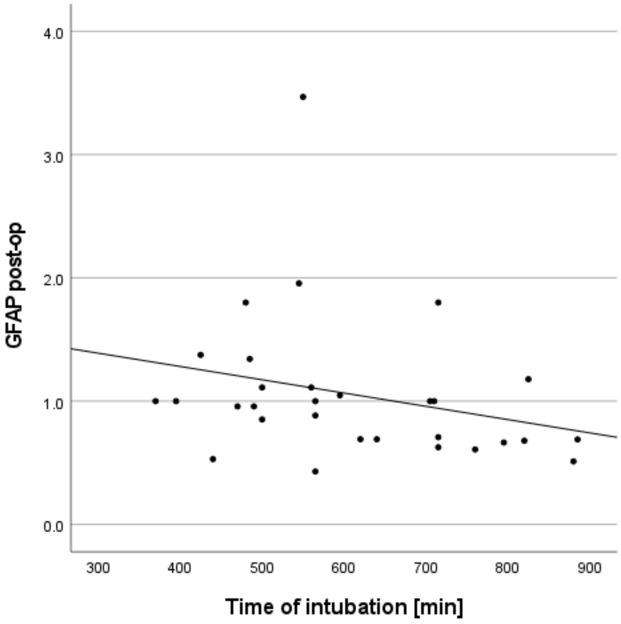

(**b**)

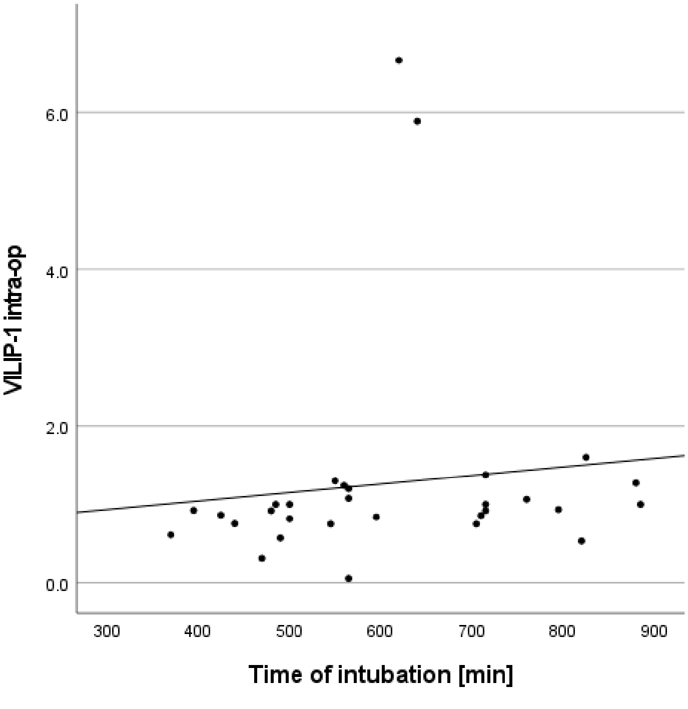

(**c**)

Figure S1. Scatter plots with fit lines. (a) Relationship between intraoperative expression of microRNA-1-3p and time of intubation (min); (b) Relationship between postoperative expression of glial fibrillary acidic protein (GFAP) and time of intubation (min); (c) Relationship between intraoperative expression of visitin-like protein 1 (VILIP-1) and time of intubation (min).

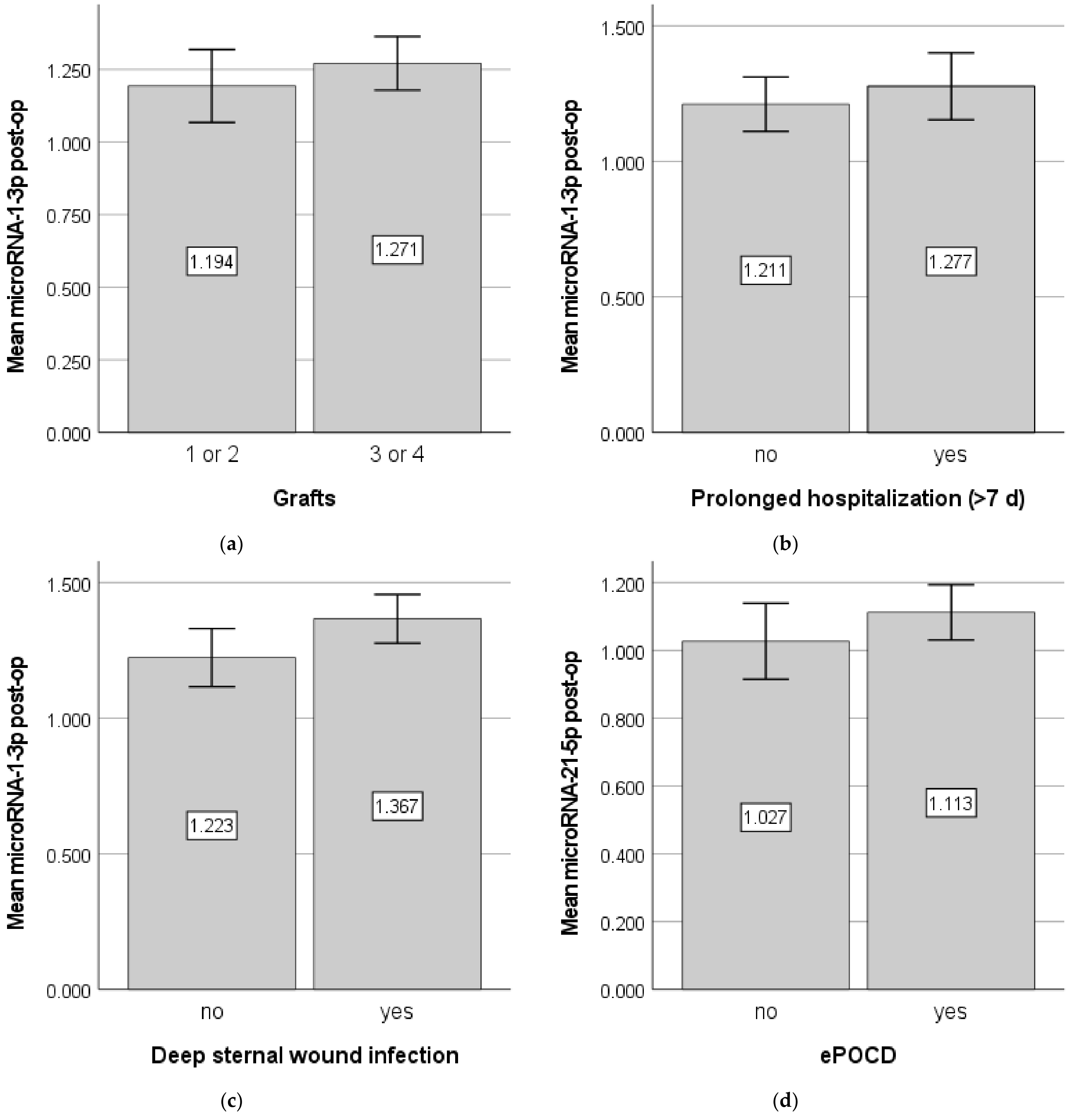

Figure S2. Bar charts comparing means in groups, with bar errors representing the variable's standard deviation in groups. (a) Postoperative expression of microRNA-1-3p in patients who received 1 or 2 grafts and 3 or 4 grafts; (b) Postoperative expression of microRNA-1-3p in patients with and without prolonged hospitalization (>7d); (c) Postoperative expression of microRNA-1-3p in patients with and without deep sternal wound infection; (d) Postoperative expression of microRNA-21-5p in patients with and without early postoperative cognitive dysfunction (ePOCD).

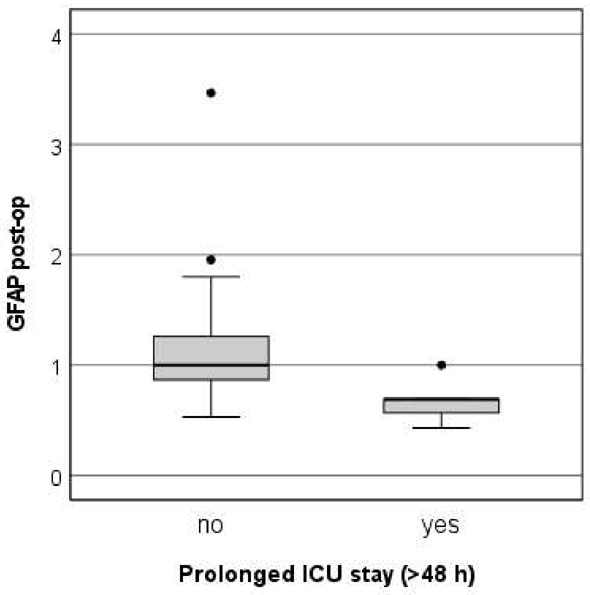
GFAP post-op
0
1
2
3
4
no
yes
Prolonged ICU stay (>48 h)

(**a**)

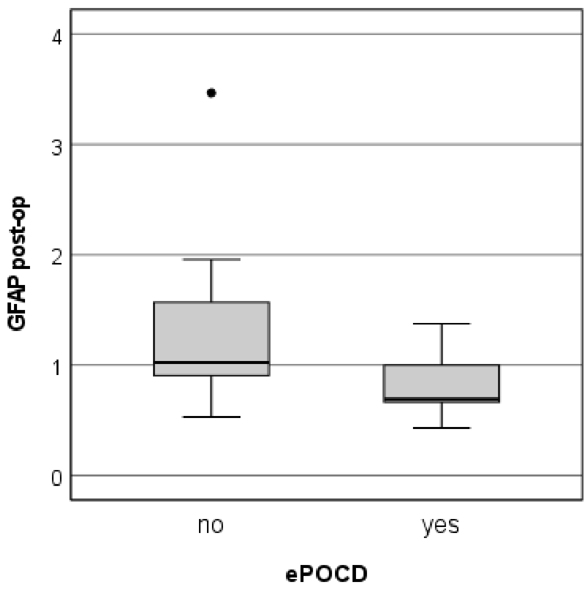
GFAP post-op
0
1
2
3
4
no
yes
ePOCD

(**b**)

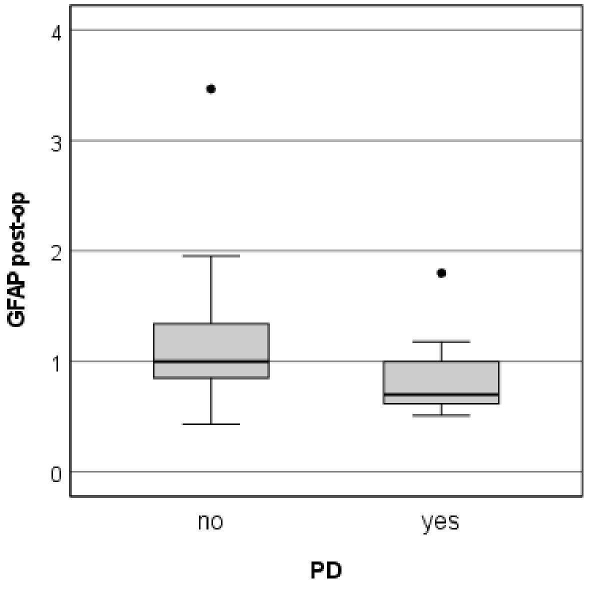
GFAP post-op
0
1
2
3
4
no
yes
PD

(**c**)

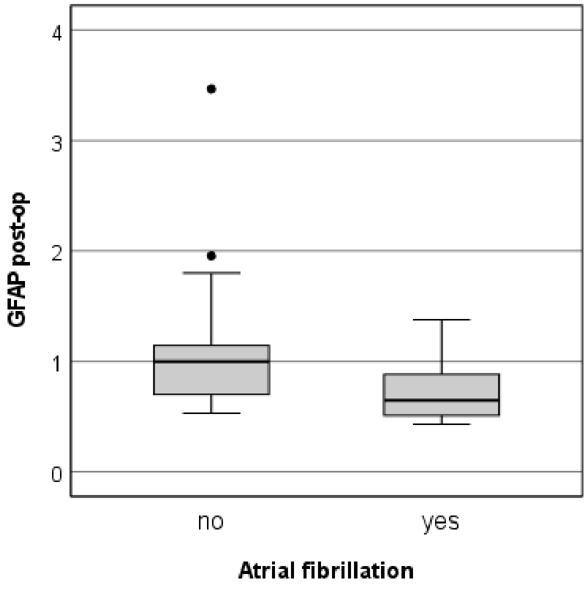
GFAP post-op
0
1
2
3
4
no
yes
Atrial fibrillation

(**d**)

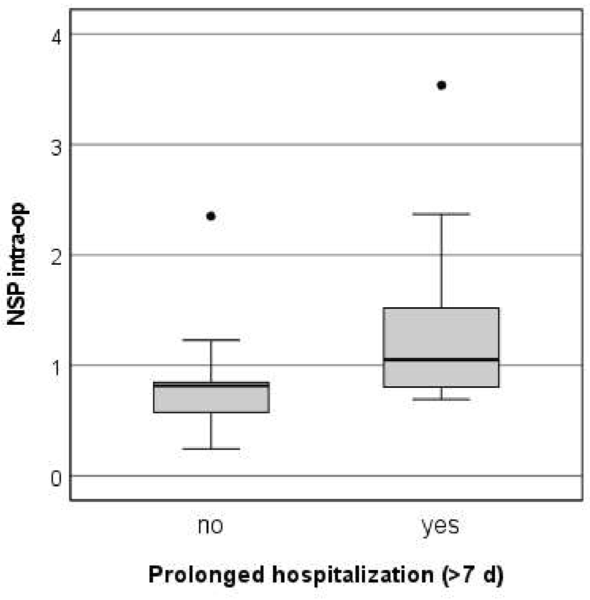

(e)

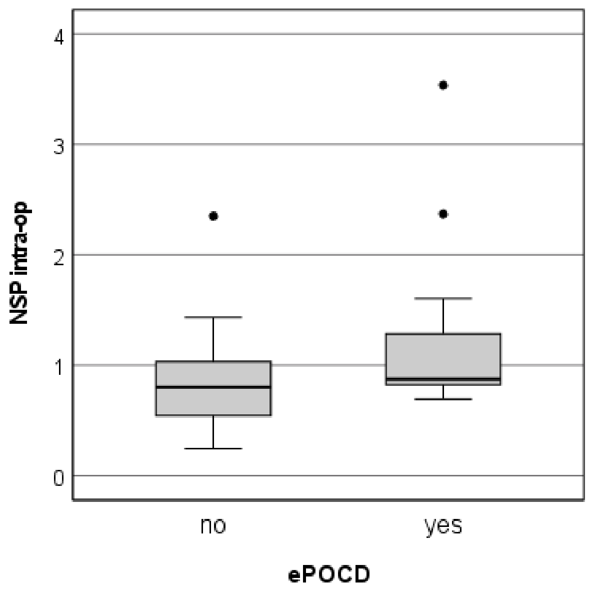

(f)

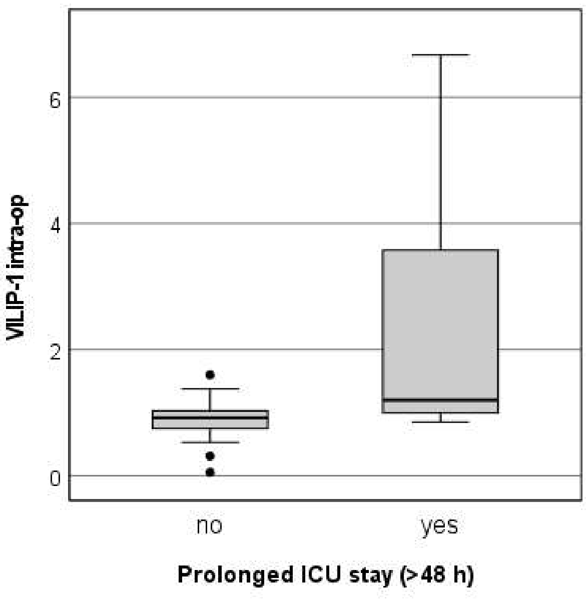

(g)

Figure S3. Box plots comparing variable's distribution in groups. The lower boundary of the box indicates the 25th percentile; the upper boundary of the box indicates the 75th percentile; the line within the box marks the median. Whiskers indicate 10th and 90th percentiles, and dots represent outliers. (a) Postoperative expression of glial fibrillary acidic protein (GFAP) in patients with and without prolonged intensive care unit (ICU) stay; (b) Postoperative expression of glial fibrillary acidic protein (GFAP) in patients with and without early postoperative cognitive dysfunction (ePOCD); (c) Postoperative expression of glial fibrillary acidic protein (GFAP) in patients with and without postoperative delirium (PD); (d) Postoperative expression of glial fibrillary acidic protein (GFAP) in patients with and without atrial fibrillation; (e) Intraoperative expression of neuroserpin (NSP) in patients with and without prolonged hospitalization (>7d); (f) Intraoperative expression of neuroserpin (NSP) in patients with and without early postoperative cognitive dysfunction (ePOCD); (g) Intraoperative expression of neuroserpin (NSP) in patients with and without prolonged intensive care unit (ICU) stay.
